# When Your Decisions Are Not (Quite) Your Own: Action Observation Influences Free Choices

**DOI:** 10.1371/journal.pone.0127766

**Published:** 2015-05-29

**Authors:** Geoff G. Cole, Damien Wright, Silviya P. Doneva, Paul A. Skarratt

**Affiliations:** 1 Centre for Brain Science, University of Essex, Colchester, Essex, United Kingdom; 2 Department of Psychology, University of Hull, Hull, Yorkshire, United Kingdom; University of Bologna, ITALY

## Abstract

A growing number of studies have begun to assess how the actions of one individual are represented in an observer. Using a variant of an action observation paradigm, four experiments examined whether one person’s behaviour can influence the subjective decisions and judgements of another. In Experiment 1, two observers sat adjacent to each other and took turns to freely select and reach to one of two locations. Results showed that participants were less likely to make a response to the same location as their partner. In three further experiments observers were asked to decide which of two familiar products they preferred or which of two faces were most attractive. Results showed that participants were less likely to choose the product or face occupying the location of their partner’s previous reaching response. These findings suggest that action observation can influence a range of free choice preferences and decisions. Possible mechanisms through which this influence occurs are discussed.

## Introduction

As social animals that interact on a daily basis, humans have evolved receptivity to one another’s behaviour, such that the actions of one individual can be influenced by the actions of others. For instance, Scheflen’s early body positioning work [1] showed that a person may unconsciously mimic the posture of others. Often referred to as the *chameleon effect* [2], other examples include the mimicking of facial expressions [3] and breathing patterns [4]. Neurological work has also examined cortical regions associated with these types of effects. For instance, functional neuroimaging studies demonstrate that finger, hand, arm, or foot movement induces activation in the corresponding areas of motor cortex in observers [5]. Using magnetoencephalography, similar findings have been reported by Haueisen and Knosche [6] in which hand-related motor cortices were activated in expert pianists when listening to piano melodies.

Although social interaction figures prominently in a range of everyday human behaviours, research examining how this can affect motor cognition has only begun to develop in the past decade or so (see [7] for a review). This ‘joint action’ work has mostly been placed within the context of action-perception processes. A number of models have now been put forward which start from the premise that rather than being separate entities, perception and action share underlying cognitive representations [8–10]. It follows therefore that one can influence the other. For instance, a leftward shift of attention will facilitate a compatible motor response (e.g., a left-handed response; [11]). More intriguing, however, is the observation that such action-perception representations also manifest during joint action. Thus, when a person observes another individual performing a response, the *perception* of this action is said to activate the functionally equivalent *response* in the observer, and can also represent various components of the response, such as the direction and location.

One notable example is the ‘joint Simon effect’ [12]. The basic Simon phenomenon refers to the shortening of response time (RT) to discriminate a stimulus that spatially corresponds to the response required [13]. Participants may, for instance, be asked to press a right-hand button when they detect a red circle and a left-hand button when they detect a green circle. Although stimulus location is task-irrelevant, results typically show that RTs are shorter when the green circle happens to occur on the left side of the display compared to when it occurs on the right. Similarly, RTs are reduced when the red circle happens to occur on the right side of the display than when it occurs on the left. Sebanz, et al. [12] undertook a joint action variation of the Simon task in which two participants sat adjacent to each other and shared responses. One observer pressed a left-hand button when one stimulus was presented, and the other observer pressed a right-hand button when another stimulus was presented. The same observers also performed their task alone. Results showed that a Simon effect occurred in the former condition but not in the latter. Although the explanation has been challenged see [14], Sebanz et al. [12] argued that although a partner’s task was irrelevant to one’s own, the observers represented each other’s stimulus-response rule.

A further example concerns an effect explored in the present work. Welsh and collaborators [15] had participants sit beside each other and take turns to reach out to one of three targets that occurred on a table top. One target could appear on the left of the participant sitting on the left, another target could appear on the right of the participant sitting on the right, and a third target could appear between the two. Results showed that participants were relatively slow to initiate a reaching response to the same target as their partner’s previous response. Furthermore, Welsh et al. [15] also showed that a person would be slower to initiate a response that was compatible to the relative direction of their partner’s previous response. That is, a participant sitting on the left would be slower to respond to their left hand target when their partner had also just made a left target response. This suggests that action observation mechanisms encode egocentric as well as allocentric response information. In line with previous work [16] the authors argued that these results were evidence of inhibition of return (IOR; [17]) in which participants are relatively slow to respond to previously attended stimuli. Thus, Welsh et al.’s [15] data suggest a slowing of responses to locations that have been acted upon by another person, an effect Skarratt and colleagues [18] have termed ‘social IOR’.

The current work examined whether action observation can influence the decisions and judgements one makes about a stimulus that appears in a location to which another person has just reached. It is now well established that supposedly ‘free choice’ decisions can be influenced by a variety of inducing stimuli. For example, Schlaghecken and Eimer [19] asked participants to freely choose whether to press a left or a right button. Before they were prompted to do so, a masked arrow prime (either a “<<” or a “>>”) was presented below the perceptual threshold. Results showed that even though the primes were not consciously perceived, participants were more likely to choose a response compatible with the direction of the prime. In a similar vein, Wilson and Pratt [20] have shown that non-social IOR can bias subsequent responses. They used the standard IOR paradigm in which a task irrelevant precue appeared in one of two spatial locations either side of a central fixation point (i.e., [17]). Upon seeing the cue, participants were required to freely choose a left or right button. Results showed that participants tended to avoid selecting the button that corresponded with the previously cued location.

**Fig 1:**
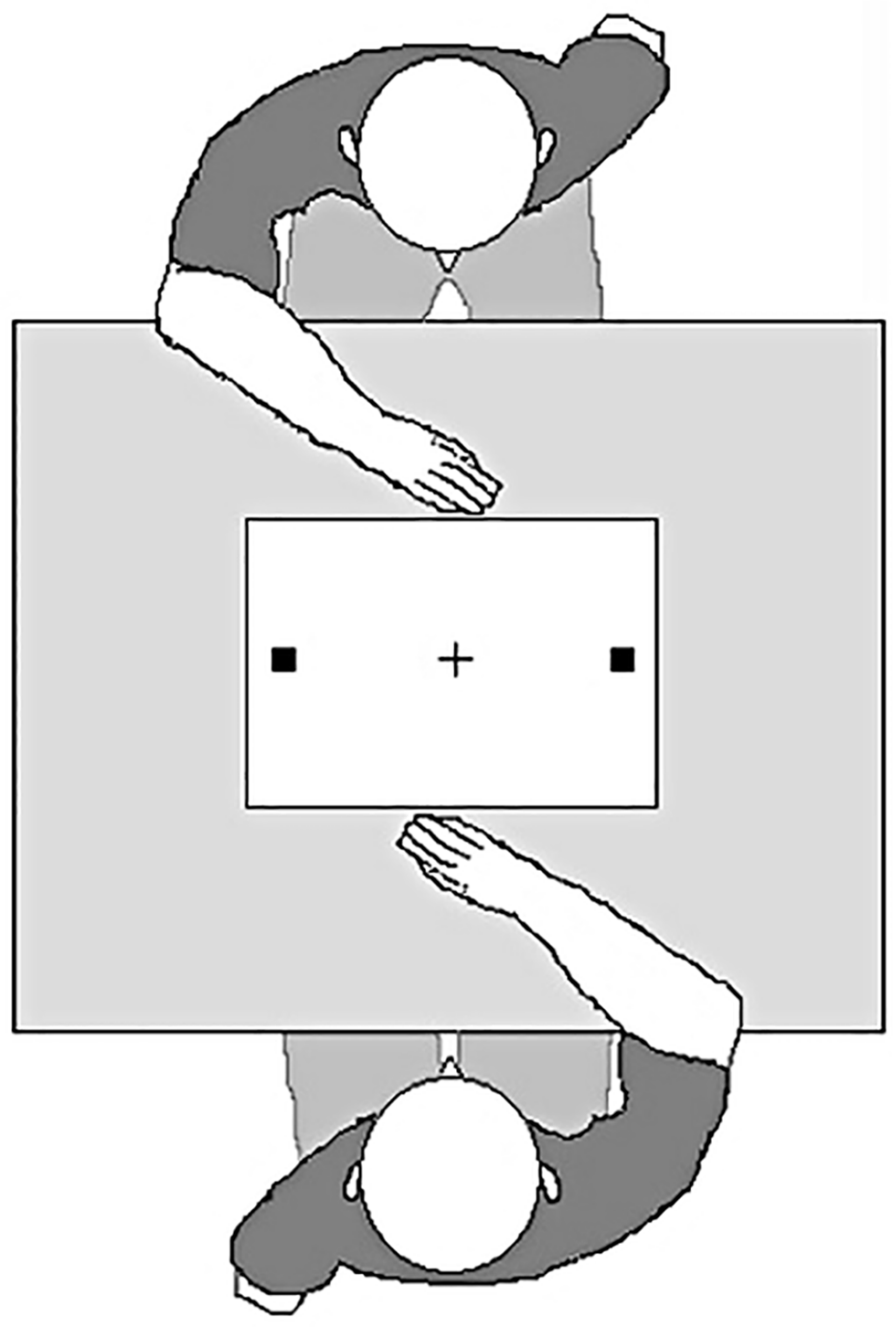
Schematic representation of the paradigm employed in the four experiments. In Experiment 1 participants take turns to freely select and reach out to one of the two squares. In Experiment 2 the squares were replaced by images of products and participants asked to decide which of the two they preferred (or least preferred). In Experiments 3 and 4 pairs of faces were presented and participants asked to decide which of the two they thought most people would consider to be most (or least) attractive.

In the present work pairs of participants performed a free choice version of an action observation task in which they sat opposite one another at a table (see Fig 1; [16], [18], [21]). Each took turns to reach out to one of two locations on the workspace positioned between them. In Experiment 1 participants were asked to freely select and reach to either of two occupant squares. In Experiment 2 they were presented with pairs of consumer products, asked to decide which they preferred (or least preferred), and indicate their choice by reaching to the product. In Experiments 3 they were presented with pairs of faces, and asked to decide which they thought was the most (or least) attractive and to indicate this by reaching to the face. In a fourth experiment each participant judged the attractiveness of faces with an experimental confederate rather than another naive participant. Thus, over the course of the these experiments, participants were required to make a range of free choice decisions, varying from simple (‘select a location’) to more complex (‘which is the most/least attractive face?’). In each case, participants were instructed that the presence of their partner, as well as the choices they make, should have no bearing on their own task. However, if action observation can bias the free choice decisions a person makes, we should expect the location of one participant’s reaching responses to influence the choice decisions of their partner.

## Experiment 1: Participants Freely Select and Reach to a Left/Right Location

### Method

#### Ethics Statement

All experiments in the present research have been approved by the Ethics Committee of the University of Essex. The authors have received a statement of approval from the committee. All participants gave their written informed consent to take part in this research.

#### Participants

Sixteen participants (10 females) took part in exchange for course credit.

#### Stimuli and Apparatus

The stimuli were displayed on a 22-inch LCD monitor embedded into a table such that the screen was facing upwards and had a Keytec touch-screen placed flat on top. The touch-screen surface was 740 mm from the floor and participants sat with their chest approximately 240 mm from a ‘home’ button positioned directly in front of them. To account for slight differences in the height and seating positions of our participants, all stimulus measurements are given in mm rather than visual angle. The stimulus positions were denoted by squares (14 mm along each side) located 310 mm from each other. The squares were black (0.4 cd/m^2^) and acted as ‘placeholders’ in that they were present for the entire duration of a condition block. They illuminated briefly (100 ms) by turning white (76.2 cd/m^2^), thus rendering them invisible momentarily. The fixation cross was black and all stimuli were presented against a uniform white background (76.2 cd/m^2^). The experiment was driven by an RM Pentium PC running custom software that controlled stimulus generation and the recording of responses. Participants made their response by releasing a home button and touching one of the two squares.

#### Design and Procedure

The experiment measured the proportion of responses each participant made to the same location as their partner’s previous response compared with the chance value of 50%. Participants were asked to use their preferred hand to rest on the home button and to make their responses. The first response of Participant A activated the stimulus sequence after which the two participants alternated single responses for the duration of the experiment. Thus, a response on one trial by, say, Participant A also acted as the inducing stimulus for the next trial for Participant B. Each response was prompted by a cue, marked by the simultaneous illumination of both stimulus squares. Upon seeing this, the participants were instructed to decide to which of the two positions they should respond. They were told that while the speed of decision was not important they should aim to respond within a ‘second or so’ of the cue. This was stated to discourage participants from labouring their decision. Once a response was completed, 350 ms elapsed until the next target occurred. They were also asked to fixate centrally at all times until they were required to make their response, during which they were instructed to fixate the response position. Furthermore, participants were told that they could ignore their partner’s response since it was not relevant to their own decision. Each block was composed of 209 trials. Because we were primarily interested in decisions based on the observation of a previous response, the first response of a block was not analysed. Thus 104 trials were generated for each participant.

### Results and Discussion

**Fig 2:**
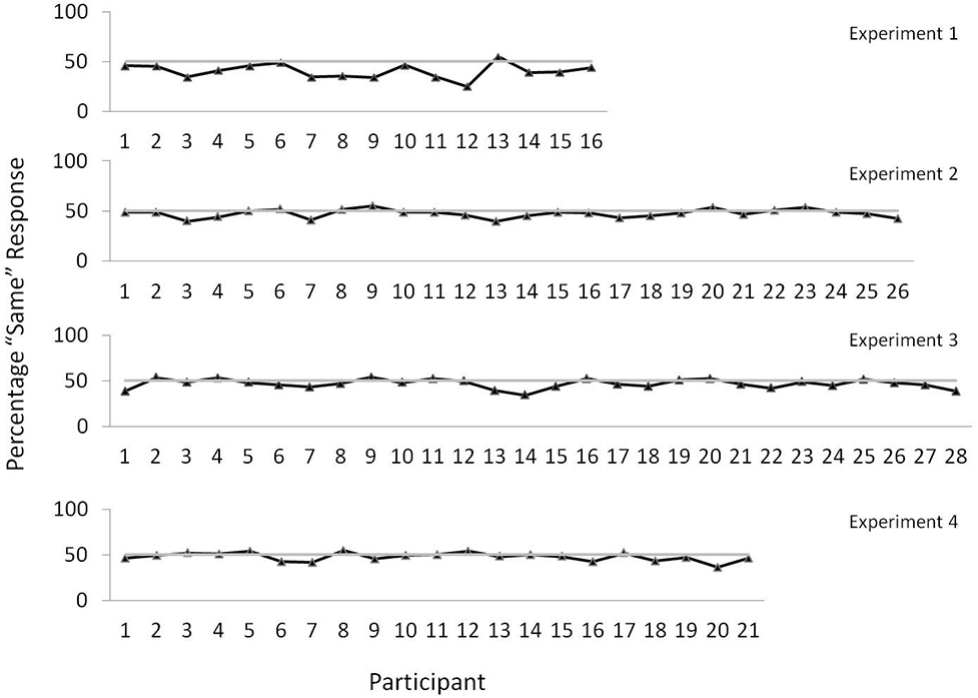
Percentage ‘same’ responses for each participant in all four experiments.

Each participant’s response was coded as being the “same” or “opposite” to their partner’s previous response. On occasions when a response was too light to be registered by the touch-screen (and its location could not be established) the response was omitted from the analysis along with the next response of the other participant. Approximately 13% of data were omitted this way. The percentage of the remaining same responses was then compared with the chance value of 50%. Results showed that overall, participants responded to the same location on 40.6% (SD = 7.41) of trials compared to 59.3% made to the opposite location (see Fig 2 for all ‘same’ responses in Experiment 1). Each person’s percentage score was entered into a one-sample t-test with 50 being the test value. This analysis showed that participants were less likely to respond to the same location than chance would allow, t(15) = 5.1, p < 0.001, Cohen’s *d* = 1.3, (95% confidence interval: lower = 37.0%, upper = 44.2%). Additionally, although participants were informed that response speed was not important we also analysed RTs. Results showed that the effect reported above did not manifest in terms of shorter RTs to reach to a different location; same RT = 537 (SD = 178), different RT = 552 (SD = 195), t(15) =. 88, p >. 39, Cohen’s *d* =. 08. Finally, we conducted one further analysis to examine whether a response strategy or bias might account for the effect (e.g., a general tendency to respond to one location more than the other). To this end, we randomly paired participants who had not performed the task together. Thus, for instance, the response from Participant number 4 on trial number 156 was followed by the response from Participant number 15 on trial number 157. This was then followed by the response from Participant number 4 on trial number 158, and so on. Results showed that responses to the same position did not differ from chance, 50.5% (SD = 6.6), t(15) =. 27, p > 0.79, Cohen’s *d* = 0.08. Overall, these data show that participants were inclined to avoid the location to which their partner had just responded.

## Experiment 2: Participants Choose Between Products

In Experiment 1 participants were tasked with choosing to which of two spatial locations they should make a response. This represented a relatively simple decision that required very little deliberation. In Experiment 2 we examined whether one person’s actions could influence their partner’s decisions when the two spatial locations contained objects that required a more considered choice. Using the same action observation paradigm employed in Experiment 1, participants were shown pairs of familiar consumer products and asked to indicate, via a reaching response, which of the two they preferred (or least preferred).

### Method

All aspects of the method were as reported previously with the following exceptions. Twenty six participants (16 females) took part in exchange for £6. None had taken part in Experiment 1. Fifty-two pairs of familiar everyday products were generated. Examples included clothing, watches, furniture, music and gardening equipment, kettles, cars, cycles, and kitchen appliances. The pairs were randomly selected from the same category, e.g., two watches, two kettles, etc., and presented onscreen in the locations occupied by the two square cues in Experiment 1. That is, each was centred 155 mm to the left or right of a centrally positioned cross. The images of the products were all scaled to fit within an imaginary square measuring approximately 65mm along each side, and were presented in their natural colours. The trial types were presented in a random order with the stipulation that the same pair could not occur on the following trial. Thus participants were never presented with the same two items that their coactor had just made a judgement on. Participants were told that they should ‘scan both products and decide which of the two you prefer’. In a second block of trials participants were asked to decide which of the two was least preferred. The presentation order of the two blocks was counterbalanced. Each participant was presented with the same pairs of products four times over the course of the experiment, twice when judging which was the most preferred, and twice when judging which was the least preferred. Participants were also told to firmly press the touch-screen in an attempt to ensure all touch responses were recorded. There were 209 trials in each block generating 418 trials for the entire experiment.

### Results and Discussion

Of all responses only two were not registered by the touch-screen. As with Experiment 1 we compared the proportion of responses made to the same location as a partner’s previous response with the chance value of 50%. Results showed that, overall, participants responded to the same location on 47.6% (SD = 4.1) of trials (see Fig 2 for all ‘same’ responses in Experiment 2). A one-sample t-test revealed this to represent significantly fewer responses than chance would allow, t(25) = 3.0, p <. 006, Cohen’s *d* =. 58, (95% confidence interval: lower = 46.0%, upper = 49.2%). A further analysis revealed that there was no difference in this effect as a consequence of making a preferred or non-preferred decision (preferred = 47.2%, non-preferred = 48.0%), t(25) =. 54, p >. 59, Cohen’s *d* = 0.14. Furthermore, as with Experiment 1 the decision effect did not manifest in terms of RT; same RT = 1323 (SD = 238), different RT = 1305 (SD = 251); t(25) = 1.4, p >. 17, Cohen’s *d* =. 07. We also repeated the analysis we undertook in Experiment 1 to establish whether a response bias could account for the result. Data from different combinations of participants were randomly paired together. Again, responses to the same position did not differ from chance, 50.4% (SD = 6.6), t(25) =. 45, p > 0.66, Cohen’s *d* = 0.1. Overall, these data show that, in addition to the simple location choices that were measured in Experiment 1, the observation of another person’s actions can also influence more complex subjective choices; namely, preference for consumer products.

## Experiment 3: Participants Choose Between Faces

Experiments 1 and 2 represented two kinds of free choice decisions that ranged from simple (‘select a location’) to more considered (‘select your most/least preferred product’). Both types of decision were therefore based on personal preference. In Experiment 3 we explored the generality of the action observation effect by examining whether seeing a response could influence a relative judgement concerning physical attractiveness. Using the same paradigm, participants were presented with pairs of faces and asked to decide which of the two would be considered the most (or least) physically attractive by the majority of people. As before, they indicated their decision with a reaching response.

### Method

All aspects of the method were as reported in Experiment 2 with the following exceptions: Sixteen of the 28 participants were female, and none had taken part in Experiments 1 or 2. The stimuli were colour photographs of male or female faces taken from a library of images held at the University of Essex. The images were cropped to show the face with only a minimal amount of hair. Half the faces were male and half female but the pairs always comprised faces of the same sex. Prior to the experiment, an observer who was naive to the rationale and aims of the experiment was asked to sort the faces into pairs that were of similar attractiveness until 52 face pairs were generated. Thus, each participant was presented with the same pairs of faces four times over the course of the experiment, twice when judging which was the most attractive, and twice when judging which was the least attractive. Participants were asked to ‘scan both faces and decide which one you think most people would consider the more physically attractive’. In a second block participants were asked to decide which of the two they thought would be considered as the least attractive.

### Results and Discussion

All reaching responses were registered by the touch-screen. Again, we compared the proportion of responses to the same location as a partner’s previous response with the chance value of 50%. This analysis showed that participants responded to the same location on 46.6% (SD = 5.1) of trials (see Fig 2 for all ‘same’ responses in Experiment 3). A one sample t-test revealed this to be significant, t(27) = 3.5, p <. 002, Cohen’s *d* =. 66, (95% confidence interval: lower = 44.7%, upper = 48.9%). As with Experiment 2, we also analysed responses with respect to the specific judgement (i.e., most attractive, least attractive). This again showed no reliable effect of decision type, with ‘most attractive’ accounting for 45.1% of same responses, and ‘least attractive’ accounting for 48.1%, t(27) = 2.0, p >.05, Cohen’s *d* =. 46. There was also no RT effect; same RT = 1300 (SD = 494), different RT = 1294 (SD = 528), t(27) =. 37, p >. 71, Cohen’s *d* =. 01. Finally, the random-pairs analysis we performed in Experiments 1 and 2 again showed that same responses did not differ from chance, 49.3% (SD = 3.1), t(27) =. 27, p > 0.27, Cohen’s *d* = 0.23. In sum, these results show that judgements relating to the physical attractiveness of faces are affected by another person’s response position. These data thus concur with those from Experiments 1 and 2.

## Experiment 4: Participants Co-Act with an Experimental Confederate

The results of Experiments 1–3 suggest that participants tend to avoid selecting stimuli occupying the same location as their partner’s previous choice. In Experiment 4 we examine the possibility that a response strategy or bias underlies the effect we attribute to action observation. It is of particular importance to examine this issue in the present context because certain biases, for instance a tendency to respond to one side of the display, could generate a pattern of data that resembles the basic phenomena we observed. For instance, if both participants happen to have a response bias to their right (or both to their left), this would appear as if they are avoiding each other’s previous location. Indeed, although no significant laterality bias was present in Experiments 1 and 3, there was in Experiment 2 (Experiment 1: 49.9% of responses to the left, t(15) = 0.04, p > 0.96; Experiment 2: 53.4% of responses to the right, t(25) = 4.6, p < 0.01; Experiment 3: 51.9% of responses to the right, t(27) = 1.0, p > 0.32). Recall that we examined whether a bias could explain the action observation effect in Experiments 1–3 by conducting post-hoc analyses on data derived from randomly combining participant pairs. Although the results suggest the bias account does not explain the effect, the present experiment offered a direct test of this possibility. Experiment 4 was a close replication of the method employed in Experiment 3 with the exception that one of the observers was a confederate (the third author) whose responses were predetermined such that she made an equal number of left and right responses. This approach is analogous to a standard visual attention experiment in which ‘cues’ are equally distributed between left and right, a design that controls for any issues associated with response bias. If a participant demonstrates a bias to localize targets on one side of the array, their accuracy will be at chance. Similarly, if our participants strategically bias their responses to one side of the array, their proportion of same-location responses will be 50%.

### Method

All aspects of the method were as reported in Experiment 3 with the exceptions that 21 participants (16 females; all right-handed) performed with the experimenter posing as their coactor. A small grey square appeared in a corner of one side of the display together with the two faces. This informed the confederate which of the two locations should be responded to. When asked after the experiment, none of the participants stated that they saw the square. Because the basic effect in Experiments 2 and 3 did not differ according to type of decision (e.g., preferred or non-preferred), participants in Experiment 4 only performed one block of trials in which they were required to reach to the most attractive face.

### Results and Discussion

Only one response was not registered by the touch-screen. Results showed that participants were less likely than chance to choose the face located in the position that the confederate had just responded to (47.7%; SD = 4.8; see Fig 2 for all ‘same’ responses in Experiment 4). A one sample t-test revealed this to be significant, t(20) = 2.2, p <. 038, Cohen’s *d* =. 49, (95% confidence interval: lower = 45.7%, upper = 49.8%). Again, there was no effect of RT; same RT = 1054 (SD = 212), different RT = 1053 (SD = 232); t(20) =. 13, p >. 89, Cohen’s *d* <. 01. There was however a bias towards reaching to the right (67.3% of responses to the right, t(25) = 6.0, p < 0.01). In sum, these findings replicate the effect we saw in Experiment 3 concerning facial attraction and more generally the results from Experiments 1 and 2.

Finally, there has been a debate in the past 10 years or so concerning whether and how proportional data should be transformed [22]. Even before this debate started the consensus was that transformed data is not required when all values are between. 3 and. 7. Although none of the participants generated proportions beyond. 3 and. 7 in any of our four experiments, we did perform additional analyses using arcsine transformed proportions. The basic response effect we report in each experiment was still observed.

## General Discussion

Recent work suggests that the response behaviour of one person can influence the subsequent response of another. For instance, Welsh et al. [16], [23] and Skarratt et al. [18] demonstrated that observing another individual reaching to a location slows an observer’s response to the same location. In the current work we asked whether one person’s reaching response can influence an observer’s free choice decisions. Our results suggest that this is indeed the case. The overarching finding is that a person is less likely to choose an item that occupies the same spatial location as one previously selected by another person. This basic finding holds true for a range of free choice decisions, varying from simple to more complex: Not only do people tend to avoid repeating the response location of their partner (Experiment 1), their partner’s response position also influences their personal preference for consumer products (Experiment 2) and judgments concerning the physical attractiveness of faces (Experiments 3 and 4). Thus, the common factor in all these free choice decisions is the behaviour of another person. As such, these findings extend what is currently known about joint action effects; the observation of another person’s response can influence processes occurring at relatively late cognitive stages, i.e., free choices, decisions, and the judgements one makes. In a standard joint action experiment pairs of participants respond to a location determined by the position of a target. In the present experiment by contrast, participants freely chose their response position. These findings are also consistent with other work showing that basic visuomotor processes can bias simple localisation responses [19], [20]. However, the current findings suggest that more considered subjective judgments, such as those indicating a preference between two alternative products or faces, are also susceptible to their influence.

The present findings clearly raise the question of what mechanism(s) may be responsible. One possibility is social IOR [16], [18], [21]. IOR is the biasing of further attentional and motor resources being allocated to recently inspected objects and locations, and can therefore account for why individuals tend not to revisit spatial locations selected by their partner. In the present context, one person’s reaching response is likely to have elicited in their partner an attentional shift plus associated motor programming to the response location. By the time the partner themselves are required to make a decision on the next trial (350 ms inter-trial interval; see Method) the stimulus located at the responded-to location may still be subject to inhibitory effects. If the mediating mechanism is indeed IOR, then it presents some intriguing questions about the specific nature of the responses observed in our experiments. For instance, which processes are inhibited in social IOR, and how do they give rise to biased decision making? If, in accordance with IOR there are perceptual and motor components to the inhibition, does the observation of another person’s response impair the perceptual representation of an item subsequently appearing in that location, or bias motor programming away from its location, or result in some combination of the two? The corollaries of these possibilities are that participants tend to avoid choosing items with a relatively degraded perceptual representation, and/or which require the programming of successive motor responses.

A related suggestion is that the generation of social IOR shifts the valuation weightings between inhibited and non-inhibited items. The *gaze-cascade model* [24] provides a means by which this could occur. The model states that rather than being one outcome of a person’s preference for an item, a person’s gaze is integral in the formation of that preference. This is based on findings showing that participants increase their fixations of a stimulus in the moments prior to selecting it as their preferred choice, an effect known as gaze bias [24]. Hence, any tendency to direct gaze away from a partner’s previous response location—and consequently to a novel location—may force preference formation towards the non-inhibited item. Indeed, IOR does seem to have a significant oculomotor component [25], leading to the intriguing possibility that participants simply prefer the items they are biased to look at. However, two aspects of the present data do not concur with the basic social IOR effect and thus the general IOR explanation. Welsh et al. [16], [23] have shown that participants are not only slower to reach to a location that another individual has just responded to but are also slower to repeat their own action relative to their last response, what the authors called ‘within-participant IOR’. Additional analysis on all the present experiments revealed that participants did not reliably avoid their previous response position, i.e., they only did so on only one of the four experiments: (Experiment 1; Same response = 41.7%; SD = 10.6, t(15) = 3.1, p <. 01, Cohen’s *d* =. 78. Experiment 2; Same response = 50.6%; SD = 7.7, t(25) =. 43, p >. 67, Cohen’s *d* =. 08. Experiment 3; Same response = 53.4%; SD = 7.0, t(27) = 2.6, p <. 05, Cohen’s *d* =. 49. Experiment 4; Same response = 51.5%; SD = 7.1, t(20) =. 97, p >.35, Cohen’s *d* =. 21). Furthermore, the absence of an RT effect in all our experiments also fails to support the IOR account, although this may be due to the fact we did not emphasise speed of response. Nevertheless, although these two aspects of the data do not support the strictest version of the IOR account, they offer a starting explanation of why participants tended to avoid responding to the same location as their coactor.

If social IOR does indeed explain the free choice bias observed in the present study, our findings in turn challenge one possible account of the basic social IOR effect. In the standard paradigm participants alternate responses to the location of a target that occurs in one of two positions. In line with other action observation work, both Welsh et al. [16] and Skarratt et al. [18] argued that this effect was due to the joint representation of observation and response. However, an alternative explanation is that the effect is due to a relatively high-level decision process known as the ‘gambler’s fallacy’ [26]. It is well established that humans are poor at generating random choices/responses [27]. In particular, they often fail to produce long runs or use all available numbers [28] and avoid repeating digits when asked to generate a random binary sequence. The gambler’s fallacy is one expression of this inability to produce random sequences. It is a tendency to believe that a repeat is less likely to occur than probability theory actually predicts and has been shown to occur in free-choice paradigms [29]. In the context of the standard social IOR experiment observers may implicitly assume that the upcoming target is less likely to appear at the same location relative to the opposite location. That is, they may be committing the gambler’s fallacy. This could manifest itself in terms of reduced response preparation to a repeated target and hence slower RTs. The present findings undermine the gambler’s fallacy account as participants freely chose their response location rather than anticipate a future target location for their response. It is however still possible that some form of the gambler’s fallacy was operating. Participants may have subconsciously (or consciously) tried to evenly distributed responses between the two positions. Thus, if one’s partner had just responded to the left position, a participant may have then felt the need to go to the opposite location.

An alternative explanation of the present findings is the action corepresentation/imitation account. Some authors refer to the basic ‘social IOR’ effect as an ‘action congruency’ phenomenon [30] and place it within the context of perceptual-motor models [8]-[10]. As noted in the Introduction, the basis of these models is that perception and action share cognitive representations. Consequently, the observation of an individual’s action is said to activate the motor system of the observer. Corepresentation of perceived and performed actions has received an abundance of support (see [31] for a review). For example, observing a movement congruent with one’s own response facilitates the movement [32]. In the present experiments therefore, participants may have been primed to imitate an action they had just observed within an egocentric framework. For instance, seeing one’s partner reach to their left may have induced imitation such that the observer had a propensity for performing the same action, that is, reach to their left.

One further interesting aspect of the present findings concerns the size of the basic effect in Experiments 2–4 compared to Experiment 1. As could be seen in Fig 2 in the first experiment participants chose the same position that their coactor had just responded to on 40.6% of responses compared to 47.6, 46.6, and 47.7 in the last three experiments respectively. A major difference between Experiments 1 and 2–4 is that in the latter participants were required to make a more considered decision concerning a pair of stimuli. In Experiment 1, by contrast, participants were only required to choose one of two locations. Therefore, in Experiments 2–4 the processes that generate the action observation effect would have been competing against higher order decision processes. For instance, in the product choice experiment (Experiment 2) there would presumably have been many trials in which participants had a definite preference for which product they preferred. Here, it is unlikely that action observation processes would have been able to override such preferences. However, on those trials where participants did not have a strong preference, the influence of the action observation processes would now be able to exert their effect on the action/location chosen. In Experiment 1 by contrast, participants probably never had a strong preference for one location over the other, thus resulting in a greater action observation effect.

In sum, we have shown that the behaviour of another person can influence which of two spatial locations a person will freely select. Two possible explanations concern evolved inhibitory mechanisms that bias a person’s attention and/or motor programming away from locations and action corepresentation. Whichever of the hypothesised mechanisms might be responsible, however, all have marked consequences for the notion of free choice. A person may not be able to make a fully informed decision about their choices, as their visuomotor systems have restricted their available options.
